# Supplementary material for: Complexome profiling of the Chlamydomonas psb28 mutant reveals TEF5 as an early PSII assembly factor
Source: Plant Cell. 2025 Mar 17;37(6):koaf055. doi: 10.1093/plcell/koaf055 (PMC12164590; doi:10.1093/plcell/koaf055)
Supplement: koaf055_Supplementary_Data [file koaf055_supplementary_data.zip › psb28_interactive.zip!/plots/intensity/qualityPlots/peptides/HSP70C_Cre09.g393200.t1.2.html]

### Cre09.g393200.t1.2

Trivial name: HSP70C  
SeqCoverage [median]: 56.00  
Mass: 70.67 kDa  
  
 

| Sequence | MissedCleavage | Mass [Da] | wtIntensity | muIntensity | Score |
| --- | --- | --- | --- | --- | --- |
| AASQSEDLPDLK | 0 | 1272.62 | 6495.97 | 15161.73 | 253.10 |
| AASQSEDLPDLKAK | 1 | 1471.75 | 4270.15 | 6779.12 | 116.84 |
| AKLELLVK | 1 | 912.60 | 0.00 | 5343.61 | 95.50 |
| DAETYAEK | 0 | 925.40 | 0.00 | 322.35 | 73.67 |
| DAETYAEKDK | 1 | 1168.52 | 7445.68 | 15565.33 | 105.52 |
| DAETYAEKDKTR | 2 | 1425.67 | 1097.10 | 0.00 | 90.61 |
| DAETYAEKDKTRK | 3 | 1553.77 | 1333.05 | 3769.54 | 122.45 |
| DAGVQPK | 0 | 713.37 | 1963.35 | 5510.39 | 124.34 |
| DIQEVLLVGGMTR | 0 | 1429.76 | 13002.59 | 53161.05 | 204.85 |
| DKATGKEQSVR | 2 | 1217.64 | 818.21 | 3188.87 | 106.67 |
| DPSKGVNPDEVVAMGAAIQGGVLR | 1 | 2379.22 | 0.00 | 1418.36 | 89.66 |
| ELIEAK | 0 | 701.40 | 2431.36 | 7436.38 | 109.70 |
| ELIEAKNEADTAIYTTEK | 1 | 2038.01 | 11018.66 | 27907.93 | 195.97 |
| ESGIDLSK | 0 | 847.43 | 0.00 | 4869.64 | 104.44 |
| ESGIDLSKDR | 1 | 1118.56 | 23512.70 | 45302.61 | 84.69 |
| EVFQRDPSK | 1 | 1104.56 | 890.35 | 3691.48 | 71.35 |
| GVNPDEVVAMGAAIQGGVLR | 0 | 1952.01 | 2101.04 | 17024.96 | 108.45 |
| GYDDPQTQK | 0 | 1050.46 | 4351.45 | 7025.93 | 162.97 |
| GYDDPQTQKEAK | 1 | 1378.64 | 8392.18 | 4818.53 | 113.65 |
| IGETLAQQSGSSSSSSSSSSGSSDSGSSSSEEKK | 1 | 3225.39 | 9436.73 | 25481.43 | 122.57 |
| IINEPTAAALAYGTDK | 0 | 1646.85 | 0.00 | 1307.44 | 80.69 |
| IINEPTAAALAYGTDKK | 1 | 1774.95 | 25782.31 | 115703.68 | 126.00 |
| IQALSTASMK | 0 | 1048.56 | 470.65 | 10330.46 | 99.86 |
| IQSSGGLSDDQINQMVR | 0 | 1846.88 | 9554.47 | 28531.39 | 262.30 |
| KSQVFSTAADNQTQVGIK | 1 | 1920.99 | 729.81 | 1821.30 | 83.75 |
| LIGRGYDDPQTQK | 1 | 1489.75 | 478.39 | 1638.46 | 92.86 |
| LIGRGYDDPQTQKEAK | 2 | 1817.93 | 489.16 | 1427.07 | 82.59 |
| LPQAVVDEIQK | 0 | 1238.69 | 0.00 | 12395.89 | 126.71 |
| LVGLPAKR | 1 | 852.55 | 3077.36 | 6612.93 | 80.38 |
| MKETAEAYLGHPVSK | 1 | 1659.83 | 16407.84 | 42499.83 | 103.60 |
| NEADTAIYTTEK | 0 | 1354.63 | 4336.20 | 26139.46 | 201.09 |
| QAVTNPTNTVYATK | 0 | 1506.77 | 4758.83 | 12853.26 | 194.17 |
| QAVTNPTNTVYATKR | 1 | 1662.87 | 12673.42 | 13626.72 | 88.39 |
| SKLPQAVVDEIQK | 1 | 1453.81 | 3721.42 | 19909.20 | 93.35 |
| SLAEYK | 0 | 709.36 | 0.00 | 8478.26 | 107.74 |
| SQVFSTAADNQTQVGIK | 0 | 1792.90 | 12678.22 | 29180.80 | 186.16 |
| TTPSVIAFTDKGER | 1 | 1520.78 | 33979.43 | 96549.49 | 120.84 |
| VFQGER | 0 | 734.37 | 4434.33 | 7782.65 | 109.88 |
| VFQGEREMAADNK | 1 | 1493.69 | 1605.87 | 5186.92 | 64.65 |
| VIENAEGAR | 0 | 957.49 | 36885.93 | 63063.39 | 187.82 |
| VNEIVK | 0 | 700.41 | 7374.90 | 18441.20 | 148.21 |
| VNEIVKEVFQR | 1 | 1359.75 | 2360.02 | 8550.27 | 107.06 |
| VNEIVKEVFQRDPSK | 2 | 1786.96 | 0.00 | 1716.18 | 85.91 |
|  |  |  | 280359.1 | 787525.5 |  |
